# Distribution and Nucleotide Diversity of *Yr15* in Wild Emmer Populations and Chinese Wheat Germplasm

**DOI:** 10.3390/pathogens9030212

**Published:** 2020-03-13

**Authors:** Yu He, Lihua Feng, Yun Jiang, Lianquan Zhang, Jun Yan, Gang Zhao, Jirui Wang, Guoyue Chen, Bihua Wu, Dengcai Liu, Lin Huang, Tzion Fahima

**Affiliations:** 1 State Key Laboratory of Crop Gene Exploration and Utilization in Southwest China, Sichuan Agricultural University, Wenjiang 611130, China; s20173054@stu.sicau.edu.cn (Y.H.); zhanglianquan1977@126.com (L.Z.); jirui.wang@gmail.com (J.W.); guoyuech74@hotmail.com (G.C.); wubihua2017@126.com (B.W.); dcliu7@yahoo.com (D.L.); 2 Triticeae Research Institute, Sichuan Agricultural University, Wenjiang 611130, China; b20161014@stu.sicau.edu.cn; 3 College of Agronomy, Sichuan Agricultural University, Wenjiang 611130, China; 14278@sicau.edu.cn; 4 Biotechnology and Nuclear Technology Research Institute, Sichuan Academy of Agricultural Sciences, Chengdu 610061, China; 5 Key Laboratory of Coarse Cereal Processing, Ministry of Agriculture, School of Pharmacy and Bioengineering, Chengdu University, Chengdu 610106, China; yanjun62@cdu.edu.cn (J.Y.); zhaogang@cdu.edu.cn (G.Z.); 6 Institute of Evolution, University of Haifa, 199 Abba-Hushi Avenue, Mt. Carmel, 3498838 Haifa, Israel; tfahima@univ.haifa.ac.il

**Keywords:** stripe rust resistance, *Yr15*, nucleotide diversity, wild emmer populations, Chinese wheat germplasm

## Abstract

Stripe rust, caused by *Puccinia striiformis* f. sp. *tritici* (*Pst*), is a devastating fungal disease of wheat. The wild emmer gene, *Yr15* (*Wtk1*), which confers a strong broad-spectrum resistance to *Pst* isolates, is composed of kinase and pseudokinase domains. The analysis of 361 wild emmer accessions from a wide range of natural habitats confirms that functional *Wtk1* is distributed mainly along a narrow axis from Mt. Carmel to Mt. Hermon regions, in the northern part of Israel, where environmental conditions are favorable to the onset of stripe rust. An analysis of full-length *Wtk1* DNA sequences from 49 wild emmer accessions identified three haplotypes and extremely low nucleotide diversity (π = 0.00002). The sequence diversity of *Wtk1* is 9.5 times lower than that of broad-spectrum partial resistance gene *Yr36* (π = 0.00019), and both are in sharp contrast to the high level of nucleotide diversity previously reported for race-specific resistance genes (e.g., *Lr10* and *Pm3*). However, the nonfunctional *wtk1* sequences possess high level of nucleotide diversity (π = 0.07). These results may reflect the different resistance mechanisms and the different evolutionary processes that shaped these resistance genes. *Yr15* was absent in 189 Chinese wheat landraces and was present in only 1.02% of the 583 tested modern Chinese wheat cultivars. These results corroborate our previous results showing that *Yr15* was absent in 94% of a worldwide collection of 513 wheat cultivars, therefore indicating the importance of *Yr15* in wheat stripe rust resistance breeding programs in China and elsewhere around the globe.

## 1. Introduction

Wheat provides about 20% of the calories and proteins in the human diet globally [1]. *Puccinia striiformis* f. sp. *tritici* (*Pst*), the causal agent of wheat stripe rust, is one of the most devastating fungal diseases of wheat. Due to the rapid evolution of the pathogen and the emergence of new virulent and highly aggressive *Pst* races, severe yield losses occurred during recent decades in most wheat-producing areas around the globe [2]. Planting wheat cultivars with adequate levels of resistance is the most effective and environmentally friendly strategy to control stripe rust.

Based on plant growth stages, stripe rust resistance can be classified as either all-stage resistance (ASR) or adult plant resistance (APR). ASR is effective at both seedling and adult plant growth stages, whereas APR is primarily effective in later stages of plant growth [3]. In wheat, ASR is usually controlled by a single resistance gene conferring high levels of resistance to specific *Pst* races [4]. This kind of resistance is vulnerable and can be rapidly overcome by new virulent races that frequently evolve in the *Pst* populations [5,6,7]. In contrast, APR is usually quantitatively inherited and often shows a broad-spectrum resistance that is partial and, in some cases, has proven to be more durable than ASR [8,9].

In general, ASR genes encode NB-LRR proteins, which include a nucleotide binding (NB) site domain and a leucine-rich repeat (LRR) [10]. Molecular evolution and genetic diversity studies of race-specific NB-LRR genes revealed high levels of nucleotide diversity [11,12], presumably because of the arms race with pathogen effectors, which drives them to change recognition specificity frequently [13]. In contrast, APR genes, such as *Yr36* and *Lr34*, that provide partial resistance to broad spectrum of pathogen races, are highly conserved, probably because these genes interact with plant substances rather than specific pathogen effectors [14,15,16].

Different to other race-specific ASR genes, *Yr15* provides high levels of resistance to a broad spectrum of *Pst* races [17]. The *Yr15* gene is derived from wild emmer wheat (*Triticum turgidum* ssp. *dicoccoides*, 2n = 4× = 28, BBAA) [18]. Using durum wheat as a bridge, *Yr15* was introgressed into several hexaploid bread wheat lines, which were then used in several breeding programs of the world to transfer *Yr15* into prebreeding lines and commercial wheat cultivars [19]. For example, *Yr15* is recently deployed in a few commercial common wheat cultivars, such as Clearwhite 515, Patwin 515, Scarlet 09, and Summit 515 in the United States [17], but less is known about its use in wheat breeding programs in other countries, such as China. Previously, SSR diagnostic markers were developed and validated to facilitate breeding programs for incorporation of *Yr15* into wheat cultivars [19]. 

The positional cloning of *Yr15* provided us with a unique opportunity to understand the molecular evolution and genetic diversity of ASR genes conferring broad-spectrum resistance [20]. This gene encodes a tandem kinase-pseudokinase protein structure (TKP), designated as wheat tandem kinase 1 (*WTK1*), which is the first resistance gene with TKP structure discovered in wheat. The nonfunctional alleles (*wtk1*) from common wheat Chinese Spring and wild emmer Zavitan differ from the functional allele (*Wtk1*) by insertions of transposable elements, indels, and stop codons [20]. The functional allele of this gene is absent in all tested cultivated tetraploid or hexaploid wheat, except for recent introgressions [20]. Klymiuk et al. [21] demonstrated the efficiency of Kompetitive Allele Specific PCR (KASP) markers for population studies of the *Yr15* gene. However, the application of gene-specific markers should be complemented by sequence analysis to discover sequence variation and haplotypes in wild emmer populations aiming to provide a better understanding of the evolutionary processes that shaped the structure and function of *WTK1*.

The objectives of the present study were: (1) to profile the distribution of *Yr15* in wild emmer and Chinese wheat germplasm with gene-specific primers; (2) to study the sequence diversity of *WTK1* in wild emmer populations and explain its molecular evolution; (3) to characterize the stripe rust resistance in wild emmer and Chinese wheats, which carry functional *Yr15* allele.

## 2. Results

### 2.1. Distribution of Yr15 in Wild Emmer Populations 

We screened a total of 361 accessions of wild emmer using three pairs of gene-specific primers for *Yr15* gene. Accessions showing successful amplification of the expected fragment size, with at least two primer sets, were considered as *Yr15* positive. The obtained results indicated that 13.6% of the wild emmer accessions (49 out of 361) were positive for the *Yr15* gene-specific markers, while 312 (86.4%) showed no amplification for the *Yr15* gene. In total, 23 out of 108 (21.3%) collection sites in Israel included at least one *Yr15* positive wild emmer accession, whereas all the 21 accessions from Lebanon, Syria, and Turkey populations were *Yr15* negative (Table S1). 

**Figure 1:**
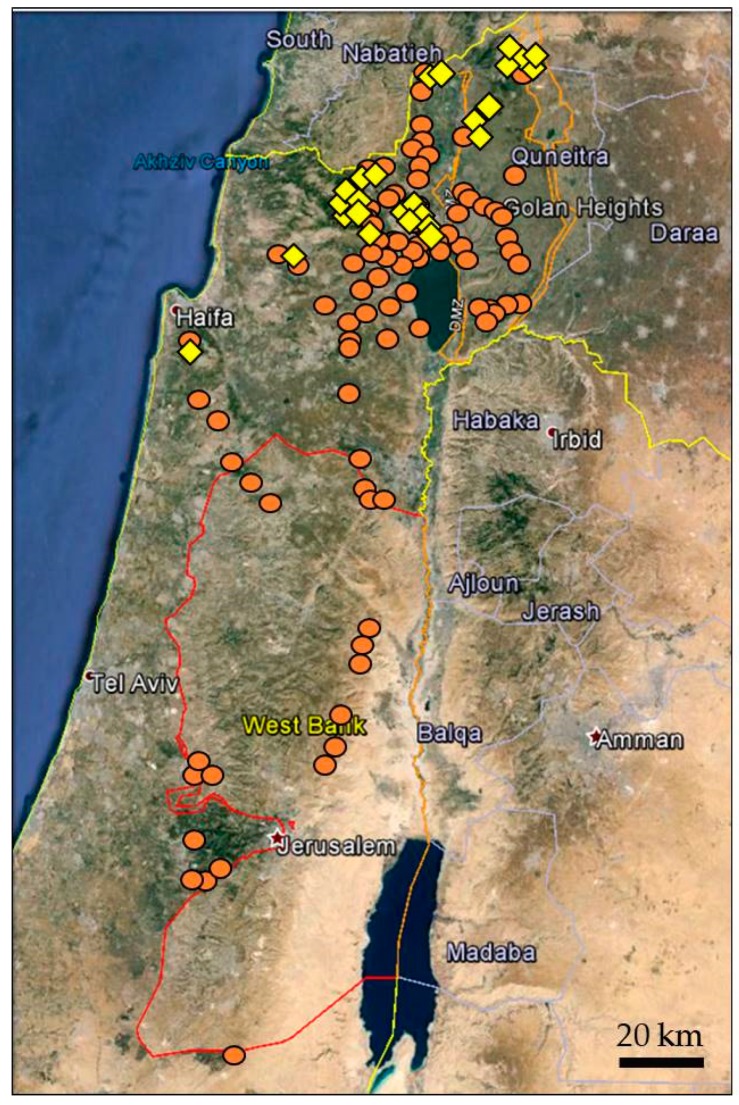
Geographic distribution of *Yr15* gene in wild emmer populations from Israel and its vicinity. Squares indicate collection sites positive for *Yr15*; circles indicate sites negative for *Yr15.*

Plotting the presence and absence of *Yr15* sites on the map of Israel shows that *Yr15* is assigned to a narrow region along an axis of ~100 km from Mt. Carmel to Mt. Hermon regions, in the northern part of Israel (Figure 1). In total, 44 out of 49 (89.8%) accessions that harbor *Yr15* were found at elevation of above 500 meters above sea level (MASL). Five accessions were collected at lower elevation (ranged from 134 to 349 MASL) in four collection sites (Elifelet, Elifelet Junc., Haggit, and Hillazon).

### 2.2. Characterization of Yr15 in Chinese Wheat Germplasm 

A large collection of 772 Chinese wheat germplasm including 189 landraces and 583 prebreeding lines/varieties in China was screened for the presence of *Yr15* using gene-specific markers. The results showed that all Chinese landraces were *Yr15* negative (Table S2), while 1.02% of Chinese wheat cultivars (six out of 583) had the *Yr15* gene (Table S3). Sequencing of full-length *Yr15* from these six wheat cultivars showed that all genomic sequences are identical to *Yr15* from G25 [20].

### 2.3. Haplotype Diversity of WTK1

**Figure 2:**
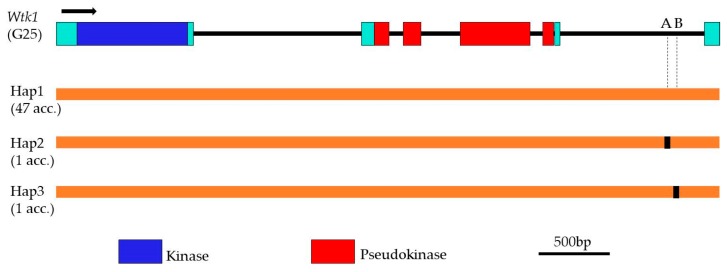
Schematic representation of the different genomic sequence haplotypes of *Wtk1* in wild emmer accessions. The haplotype of published *Wtk1* is defined as Hap1. The number of accessions corresponding to each haplotype is shown in brackets on the left. Rectangles in black represent the polymorphic nucleotides compared with *Wtk1* (G25). A, A4328G; B, C4375T; Hap 2 (GenBank MN756015); Hap 3 (GenBank MN756016).

The genomic sequence of the entire *WTK1* gene was amplified, using overlapping gene-specific primers, in 49 Israeli wild emmer accessions harboring *WTK1*. The obtained sequences were aligned against the reference sequence of *WTK1* (GenBank MG649384) [20]. Sequence alignment with the functional *Yr15* indicated that the whole region of *WTK1* is extremely conserved. Only two SNP sites (A4328G and C4375T) were detected in intron 5 among the 49 wild emmer accessions (Figure 2). SNP A4328G was detected in WEW110623 (Mt. Hermon) and SNP C4375T in WEW110846 (Meron-Kefar Shammay). *WTK1* genomic sequences from the remaining 47 accessions showed full identity to the functional *Yr15* [20]. 

Analysis of the full-length of *WTK1* in 49 wild emmer accessions identified only two SNPs defining three haplotypes with a haplotype diversity of Hd = 0.081. The published functional *Wtk1* sequence was defined as Hap 1. This haplotype was the most frequent haplotype (96%) observed. The other two haplotypes Hap2 and Hap3 of *Wtk1* were identified in WEW110623 and WEW110846, respectively.

A comparative analysis of 49 *WTK1* sequences revealed that the average number of nucleotide difference (K) is 0.082, and the overall nucleotide diversity (π) is 0.00002. 

### 2.4. Stripe Rust Resistance in Wheat Accessions That Carry Yr15

**Figure 3:**
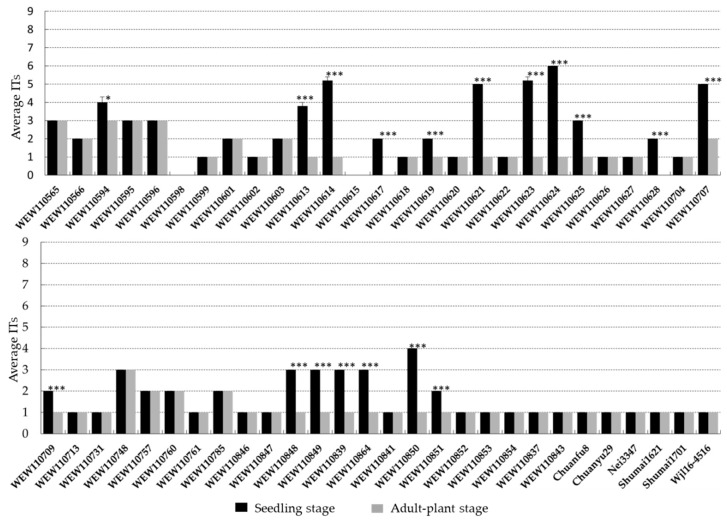
Stripe rust resistance phenotype in wheat accessions carrying *Yr15* functional allele. The stripe rust ITs of each accession at seedling and adult stages are shown on the column. Each bar represents the average value of the ITs and standard error of mean (SEM, n = 5). Some accessions showed no variation among replicates (SEM = 0). One-way ANOVA was used to determine the significance level between seedling and adult stages. Significance at *p < 0.05, significance at ***p < 0.0001.

The initial inoculation testing of 49 wild emmer accessions and six Chinese wheat cultivars that carry *Yr15* at the seedling stage showed a range of resistance responses (infection type (IT) = 0–6) against *Pst* race CYR34 (Figure 3). Of the 55 accessions, 47 (85.45%) were resistant (IT = 0–3), and eight were intermediate (IT = 4–6).

The same set of wild emmer accessions together with six Chinese wheat cultivars harboring *Yr15* were further evaluated in the field for adult-plant-stage resistance to stripe rust. All wheat accessions carrying *Yr15* showed resistance phenotype (IT = 0–3) (Figure 3). Thirty-seven accessions showed consistent IT at seedling and adult-plant stages, whereas 18 accessions had significant lower IT at adult-plant stage than those of seedling stage.

## 3. Discussion

To date, three wheat resistance genes, stripe rust resistance gene *Yr15* (*WTK1*) [20], stem rust resistance gene *Sr60* (*WTK2*) [22], and powdery mildew resistance gene *Pm24* (*WTK3*) [23], have been found to possess two tandem kinase (or pseudokinase) domains. *Yr15* and *Pm24* have been shown to confer a broader spectrum of disease resistance than *Sr60*. In the current study, we have characterized the distribution of *Yr15* gene in a large set of wild emmer wheat accessions and Chinese wheat germplasm as well as analyzed the nucleotide diversity with respect to the functional allele of *Yr15*.

### 3.1. The Geographic Distibution of Yr15 in Wild Emmer Populations

In this study, we found that a low percentage (13.6%) of wild emmer accessions from Israel (the southern distribution range of wild emmer) were *Yr15* positive, and all of the accessions from Turkey (the northern distribution range of this species) were *Yr15* negative. Our results are in agreement with those previously described by Klymiuk et al. [21]. Previous studies have shown that wheat was probably domesticated from wild emmer in southeast Turkey (northern distribution range of wild emmer) [24,25]. Recently, Nave et al. [26] demonstrated that at least part of the wild emmer from the southern region of the Fertile Crescent played an important role in the emmer domestication process. Therefore, the absence of *Yr15* gene in wild emmer accessions that reside in the northern distribution range and most of accessions in the southern distribution range, as well as in genepool of cultivated wheats, support the idea that *Yr15* was “left behind,” rather than lost, during early wheat domestication events. 

The current screening of wild emmer natural populations confirmed that *Yr15* gene is present only in northern Israeli populations and distributed along a narrow mountain ridge of about 100 km from Mt. Carmel to Mt. Hermon regions, mainly at an elevation of above 500 MASL [21]. Comparing the wild emmer collections studied here and those of in Klymiuk et al. [21], we found only 91 accessions were overlapped, while both studies revealed a similar geographic distribution of *Yr15* in wild emmer populations. It seems that selection pressure exerted by the pathogen is affecting the host-parasite interactions and co-evolution and shaping the resistance genes distribution among wild emmer populations [21,27]. In Israel, the climatic conditions are more favorable for stripe rust pathogen development in the northern regions, which are cooler and more humid during the wheat growing season, than in the southern regions of Israel [27]. Therefore, the climate conditions that favor high stripe rust pressure may exert a strong positive selection for the distribution of *Yr15* in wild emmer populations. On the other hand, wild emmer accessions are known to show a wide range of variations in flowering time due to different climate conditions within their habitats [28]. The heading date in wild emmer accessions was positively correlated with mean annual rainfall and altitude but negatively correlated with temperature variables [29]. It is known that wild emmer populations located on high mountains (high humidity and low temperature) flower later than those located on lower elevation habitats with warmer and drier climate conditions [30]. Thus, we cannot rule out the possibility that the flowering time differences in the wild emmer wheat populations are affecting the *Yr15* gene flow. 

### 3.2. Sequence Diversity of the WTK1

**Figure 4:**
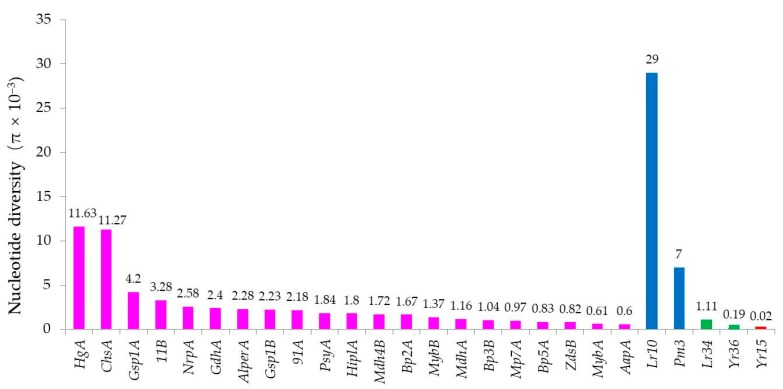
Nucleotide diversity of disease resistance genes compared with housekeeping genes in wild emmer populations. The nucleotide diversity (π) values of 21 housekeeping genes in wild emmer populations are shown in purple [31]. The nucleotide diversities (π) of race-specific genes *Lr10* [11] and *Pm3* [12] are indicated in blue, and the broad-spectrum partial resistance genes *Lr34* [34] and *Yr36* [27] are indicated in green. The nucleotide diversity of functional *Yr15* is indicated in red.

In the present study, we detected a very low nucleotide diversity of functional *Wtk1* in wild emmer populations. *Wtk1* alleles showed an average nucleotide diversity of π = 0.00002, which is approximately 38 times lower than the mean nucleotide diversity (π = 0.0027) in 21 randomly sequenced wild emmer housekeeping genes [31] (Figure 4). A similarly low level of polymorphism has been found in wild emmer gene *Yr36* (π = 0.00019) [27], which encodes a protein with a kinase and lipid binding START domain that confers partial and a broad-spectrum stripe rust resistance. In contrast, the nonfunctional *wtk1* showed a high level of nucleotide diversity (π = 0.07, calculated according to seven coding sequences of *wtk1* in wheat).

The sequence conservation of *Wtk1* is in sharp contrast to the high diversity of race-specific resistance genes, such as *Lr10* [11] and *Pm3* [12], studied in wild emmer populations. For example, the total nucleotide diversity of NB-LRR gene *Lr10* (π = 0.029) [11] in wild emmer populations was 1450 times higher than that of *Wtk1* (π = 0.00002). The sequence diversity of nonfunctional *wtk1* is comparable to those of the NB-LRR gene. The race-specific resistance genes exhibit high level of nucleotide diversity due to the co-evolution with pathogens, which drives them to change recognition specificity frequently [6,32]. In contrast, the broad-spectrum resistance genes, such as *Yr15* and *Yr36*, are expected to be more conserved. A selective sweet [33] may have shaped the very low diversity, despite allelic diversity being advantageous. Furthermore, the nature of the resistance may also result in little allelic variation being retained, despite it being advantageous. 

### 3.3. Stripe Rust Resistance Variation in Wild Emmer Accessions Carrying Yr15

In the present study, we observed a range of resistance responses in wild emmer accessions carrying functional *Yr15*. Different hypotheses may explain our observation: (1) phenotypic differences in the presence of *Yr15* are likely associated to differences in the genetic backgrounds [35]; (2) the presence of suppressors that suppressed *Yr15*-mediated resistance [36]; (3) absence/mutation of downstream substrates of resistance gene signaling pathway [21,37].

### 3.4. The Potential Value of Yr15 Gene in Wheat Stripe Rust Resistance Breeding Programs in China

In a previous study, *Yr15* was found only in the wild emmer populations and recently developed *Yr15* introgression lines but not in other cultivated wheat germplasm from around the globe [20]. In the present study, we further confirmed the lack of *Yr15* in all tested wheat landraces from ten major wheat-growing zones in China. To our best knowledge, *Yr15* has not been widely used in wheat breeding. For example, *Yr15* was just recently deployed in a few commercial common wheat cultivars in the US [17]. Zeng et al. [38] reported the absence of *Yr15* in 330 leading cultivars and 164 advanced breeding lines in China. In this study, we identified the presence of *Yr15* in six cultivars/advanced breeding lines in Sichuan province. These six lines that showed high resistance to wheat stripe rust could serve as donors for conventional introgression of *Yr15*. Among them, two leading cultivars, Chuanfu8 and Chuanyu29, were released in Sichuan in 2015 and 2017, respectively. Therefore, our results demonstrated the potential value of *Yr15* for wheat stripe rust resistance breeding in China.

## 4. Materials and Methods 

### 4.1. Plant Material

A total of 340 accessions of wild emmer collected from 108 collection sites, representing a wide range of ecogeographic distribution of wild emmer in Israel and its vicinity, and a total of 21 accessions collected from natural populations in Lebanon, Syria, and Turkey were used to profile the distribution and sequence diversity of *Yr15* in wild emmer populations. These materials were provided mainly by the Institute of Evolution Wild Cereal Gene Bank at the University of Haifa, Israel [39] and the National Small Grains collection (NSGC, USDA). The list of accessions and their collection sites are described in Table S1.

A total of 189 wheat landraces from ten major wheat-growing zones (Table S2) [40], as well as 583 wheat prebreeding lines/varieties covering most important wheat-producing regions in China (Table S3), were also included in the current study.

### 4.2. Analysis of Presence/absence Polymorphism in Yr15

Wheat genomic DNA was extracted from leaf tissue using the CTAB protocol [41]. Presence/absence of *Yr15* was detected using PCR amplification with three pairs of gene-specific primers of *Yr15* (Table S4). PCR was performed using the Gene Amp PCR system 9700 (Applied Biosystems, Foster City, CA, USA) in 20 µL reaction volume containing 1× PCR buffer, 80 ng of DNA, 300 nM of each primer, 200 µM of each dNTP, and 0.5 U of Dream Taq™ DNA Polymerase (Thermo Scientific, Waltham, MA, USA). Alternatively, the high-fidelity ExTaq polymerase (Takara, Dalian, China) was used for PCR amplifications. A touchdown PCR amplification was performed as described by Huang et al. [27]. The PCR products were visualized by 1.5% agarose gel electrophoresis, followed by staining with GoldView (Solarbio, Beijing, China).

### 4.3. Sequence Analysis of WTK1

The whole *Wtk1* gene region of over 4.6 kb was amplified from DNA samples of 49 wild emmer genotypes using four pairs of gene-specific primers (Table S4), designed based on the NCBI GenBank accession MG649384 [20]. PCR products were purified using SanPrep Column DNA Gel Extraction Kit (Sangon Biotech, Shanghai, China) and directly sequenced using Sanger chain termination method. Alternatively, purified products were cloned into pMD19-T vector (Takara, Dalian, China) and transformed into cells of *Escherichia coli* DH10B. Positive clones were identified by colony PCR. The sequencing was performed by the Sangon Biotechnology Company (Chengdu, China). Sequence reads were assembled and subjected to multiple alignments using DNAMAN software version 6.0 (Lynnon Biosoft, San Ramon, CA, USA). The obtained sequences were manually corrected. SNPs were visually checked on the chromatogram to ensure their quality. The coding sequences of *wtk1* were obtained from whole-genome assemblies of common wheat Chinese Spring [42] and wild emmer wheat Zavitan [43] as well as genomic sequence of wheat cultivars (Cadenza, Claire, Paragon, Robigus, and Kronos; https://opendata.earlham.ac.uk/wheat/under_license/). DnaSP program version 5.0 [44] was used to estimate nucleotide diversity (π) [45].

### 4.4. Stripe Rust Resistance Response

Forty-nine wild emmer accessions and six wheat varieties from Sichuan province carrying functional *Yr15* gene were tested for their response to stripe rust infection both at seedling and adult stages. The highly virulent *Pst* race CYR34 (virulent on *Yr1*, *Yr6*, *Yr7*, *Yr8*, *Yr9*, *Yr10*, *Yr17*, *Yr18*, *Yr24*, *Yr26*, *Yr27*, *Yr28*, *Yr29*, *Yr31*, *Yr43*, *Yr44*, *YrExp2*, and *YrSP*) [46] was used to inoculate the wild emmer plants and the six wheat varieties at the two-leaf seedling stage. Urediniospores used for inoculation of leaf tissue were first suspended in Isododecane and then sprayed using pneumatic airbrush (RuiYi, Guangzhou, China). Inoculated plants were placed in a dew chamber (100% humidity) at 10 °C for 16 h in the dark followed by 8 h of light. The plants were then moved to a growth chamber (75% humidity) with 10 °C during the dark period (8 h) and 15 °C during the light period (16 h). Disease severity was evaluated and characterized 14–21 days after inoculation using a 0–9 scale of infection type (IT) as described by Line and Qayoum [47]. The susceptible LDN cultivar was used as control for lack of resistance to *Pst* race CYR34. ITs were summarized by combining them into three classes of which 0–3 were considered as resistant response, 4–6 as intermediate, and 7–9 as susceptible.

A field evaluation for adult-plant stripe rust resistance of wild emmer accessions and wheat cultivars was performed at the experimental field of the Triticeae Research Institute, Sichuan Agricultural University, Wenjiang during the 2017/2018 crop season. The *Pst*-susceptible common wheat line SY95-71 was used as a spreader. Plants at the boot to heading stage were inoculated using a brush with a mixture of Chinese prevalent *Pst* races (CYR32, CYR33, CYR34, Zhong4, and HY46) mixed with talc (1:20) one hour before sunset. Disease notes were taken when susceptibility of flag leaves of SY95-71 was fully expressed.

## 5. Patents

The University of Haifa and The Natural Resources Institute Finland (Luke) had filed a patent application on the use of the sequences of the yellow rust resistance gene *Yr15* (PCT/IL2018/051081), published previously by Klymiuk et al. (2018), in which T.F. and L.H. are listed as co-inventors.

## Supplementary Materials

The following are available online at https://www.mdpi.com/2076-0817/9/3/212/s1, Table S1: Distribution of *Yr15* gene in wild emmer populations, Table S2: Chinese wheat landraces evaluated for the presence of *Yr15*, Table S3: Wheat advanced lines/cultivars in China evaluated for the presence of *Yr15*, Table S4: Oligonucleotide primers and PCR conditions used in the present study.
